# Flavonoids and Sesquiterpene Lactones from* Artemisia absinthium* and* Tanacetum parthenium* against* Schistosoma mansoni* Worms

**DOI:** 10.1155/2016/9521349

**Published:** 2016-11-17

**Authors:** Luísa Maria Silveira de Almeida, Lara Soares Aleixo de Carvalho, Matheus Coutinho Gazolla, Pedro Luiz Silva Pinto, Marcos Paulo Nascimento da Silva, Josué de Moraes, Ademar A. Da Silva Filho

**Affiliations:** ^1^Faculdade de Farmácia, Departamento de Ciências Farmacêuticas, Universidade Federal de Juiz de Fora, 36036-900 Juiz de Fora, MG, Brazil; ^2^Núcleo de Enteroparasitas, Instituto Adolfo Lutz, 01246-902 São Paulo, SP, Brazil; ^3^Núcleo de Pesquisa em Doenças Negligenciadas, Universidade Guarulhos, 07025-000 Guarulhos, SP, Brazil

## Abstract

Human schistosomiasis, caused by trematode worms of the genus* Schistosoma*, is one of the most significant neglected tropical diseases, affecting more than 200 million individuals worldwide and praziquantel is the only available drug to treat this disease.* Artemisia absinthium* L. and* Tanacetum parthenium *L. are species popularly used as anthelmintics. We investigated the* in vitro* schistosomicidal activity of crude extracts of* A. absinthium* (AA) and* T. parthenium *(TP) and their isolated compounds. AA and TP, at 200 *μ*g/mL, were active, causing 100% mortality of all adult worms. Chromatographic fractionation of AA leads to isolation of artemetin and hydroxypelenolide, while santin, apigenin, and parthenolide were isolated from TP. Artemetin, hydroxypelenolide, santin, and apigenin, at 100 *μ*M, were inactive against adult worms. Parthenolide (12.5 to 100 *μ*M) caused 100% mortality, tegumental alterations, and reduction of motor activity of all adult worms of* S. mansoni*, without affecting mammalian cells. Confocal laser scanning microscopy showed tegumental morphological alterations and changes on the numbers of tubercles of* S. mansoni* worms. This report provides the first evidence for the* in vitro *activity of parthenolide against adult worms of* S. mansoni*, opening the route to further schistosomicidal studies with this compound.

## 1. Introduction

Human schistosomiasis, caused by trematode worms of the genus* Schistosoma*, is one of the most significant neglected tropical diseases, affecting more than 200 million individuals worldwide [1]. According to World Health Organization, of the 78 countries considered endemic for schistosomiasis, only 52 countries have populations requiring preventive chemotherapy [1, 2]. In Brazil, eight million people, from endemic regions stretching from the north to the south-east of the country, mainly in the Minas Gerais State, are infected with this chronic debilitating disease [2, 3].

Although praziquantel (PZQ) is a highly effective drug for the treatment and control of schistosomiasis in mass drug administration programs, its most important limitation is the lack of activity against younger parasite stages [4, 5]. In addition, PZQ has only a limited effect on already developed liver and spleen lesions and there is a considerable concern about the development of PZQ resistance [4, 5]. Such facts have encouraged the scientific community to develop novel and inexpensive drugs against schistosomiasis [6].

In this regard, the search for antiparasitic compounds from natural sources, especially medicinal plants, has intensified [2, 7–9]. Several plants of the family Asteraceae have shown promising* in vitro* schistosomicidal activity [10, 11].

Among them, plants from the genus* Artemisia* are a rich source of bioactive sesquiterpene lactones and have a long history related to parasite control [12]. Also, several* Artemisia* species are popularly used as anthelmintics, such as* A. absinthium* L., which is known as absinthe [12, 13]. Previous studies have reported that* A. absinthium* L. presented activity against* Fasciola hepatica* [13] and* Haemonchus contortus* [14].

Also, plants of the genus* Tanacetum *(Asteraceae) are popularly used for many medicinal purposes all over the world, including anthelmintic [11].* Tanacetum *extracts and their isolated compounds are also reported to exhibit antiparasitic activities [15, 16]. Recently, our previous work has demonstrated that the crude extract and essential oil of* T. vulgare* exhibit* in vitro *schistosomicidal activity against adult worms of* S. mansoni* [11]. However, no active schistosomicidal compounds were identified from* T. vulgare*.

*Tanacetum parthenium *L., known as “Feverfew” and “Tanaceto” in Brazil, is widely used in folk medicine as anti-inflammatory and for the treatment of migraine and fever [16, 17]. Phytochemical studies have shown that* T. parthenium* contains several biologically active metabolites, mainly sesquiterpene lactones [15, 17, 18]. Several studies have also reported that* T. parthenium *extracts and its isolated compounds exhibit trypanocidal [15, 18] and leishmanicidal activities [16].

Thus, this present work evaluated the* in vitro *schistosomicidal effects of the crude extracts of* A. absinthium* L. and* T. parthenium* L., which have not yet been reported. Also, our work describes, for the first time, the schistosomicidal activity of parthenolide, the active schistosomicidal sesquiterpene lactone isolated from* T. parthenium* L.

## 2. Materials and Methods

### 2.1. Plant Material and Extraction

Leaves of* A. absinthium* L. and aerial parts of* T. parthenium *L. were collected at the Faculty of Pharmacy's Medicinal Herb Garden, Juiz de Fora city, MG, Brazil, in January, 2014. Voucher specimens of* A. absinthium* (CESJ 65106) and* T. parthenium* (CESJ 65105) were identified and stored at the Herbarium of the Botany Department of the Federal University of Juiz de Fora, MG, Brazil.

The leaf rinsed extract of* A. absinthium* L. was obtained by immersing the air-dried leaves (243 g) in dichloromethane for thirty seconds at room temperature, and the solvent was removed under vacuum below 40°C, affording 6.2 g of leaf rinse extract (AA). The crude extract AA (6.2 g) was chromatographed over silica gel (70–230 mesh, Merck) under vacuum liquid chromatography system (VLC, glass columns with 5–10 cm i.d), using hexane : EtOAc mixtures in increasing proportions as eluent, furnishing eight fractions. The resulting fractions VI (hexane : EtOAc 3 : 7; 0.95 g) and VII (hexane : EtOAc 2 : 8; 1.35 g) were submitted to column chromatography over silica gel, using CHCl_3_ : Me_2_CO in increasing proportions as eluent, followed by semipreparative reverse-phase HPLC purification (column ODS 250 × 10 mm, 5 *μ*m, UV-DAD detector at 220 nm) using MeOH : H_2_O 65 : 35 as mobile phase, furnishing the following compounds: artemetin (0.1 g; *R*_*t*_ 26.84 min.) from fraction VI and hydroxypelenolide (0.035 g; *R*_*t*_ 19.94 min.) from fraction VII.

In addition, aerial parts of* T. parthenium* (1983 g) were dried, powdered, and exhaustively extracted by maceration with ethanol : H_2_O (9 : 1 v/v). After extraction, the solvent was removed under vacuum to yield 32 g of the crude hydroalcoholic extract (TP). An aliquot of TP (15 g) was chromatographed over silica gel (70–230 mesh, Merck) using a vacuum liquid chromatography system (VLC, glass columns with 5–10 cm i.d) and hexane : EtOAc mixtures in increasing proportions as eluents, furnishing 7 fractions. Fraction IV (hexane : EtOAc 6 : 4; 2 g) was submitted to column chromatography over silica gel, using CHCl_3_-Me_2_CO mixtures, followed by semipreparative reverse-phase HPLC purification (column ODS 250 × 10 mm, 5 *μ*m, UV-DAD detector at 220 nm) using MeOH : H_2_O 75 : 25 as mobile phase, affording parthenolide (152 mg; *R*_*t*_ 7.53 min.). The resulting fractions VI (hexane : EtOAc 45 : 55; 2,2 g) and VII (hexane- EtOAc 2 : 8; 0,8 g) were submitted to column chromatography over silica gel using CHCl_3_ : Me_2_CO in increasing proportions as eluent, affording santin (335 mg, from fraction VI) and apigenin (28 mg, from fraction VII).

The chemical structures of all isolated compounds were established by ^1^H- and ^13^C- NMR data analysis in comparison to literature. The purity of the isolated compound was more than 95% based on HPLC analysis.

### 2.2. Parasite

*Schistosoma mansoni* (BH strain, Belo Horizonte, Brazil) worms were maintained in* Biomphalaria glabrata* snails as intermediate hosts and* Mesocricetus auratus* hamsters as definitive host at the Adolfo Lutz Institute (São Paulo, Brazil), according to standard procedures previously described [19]. Female hamsters, weighting 20–22 g, were infected by subcutaneous injection of 150 cercariae. After 9 weeks, adults* S. mansoni* specimens were recovered from the hamster by perfusion with RPMI 1640 medium supplemented with heparin [19]. All experiments were authorized by the Committee for Ethics in Animal Care of Faculdade de Ciências de Guarulhos (FACIG/UNIESP), in accordance with nationally and internationally accepted principles for laboratory animal use and care (CEUA, 11.794/08). The study was conducted in adherence to the institution's guidelines for animal husbandry.

### 2.3. *In Vitro* Studies with* S. mansoni*

Adult worms were washed in RPMI 1640 medium (Gibco) supplemented with 200 *μ*g/mL of streptomycin, 200 UI/mL of penicillin (Invitrogen), and 25 mM of Hepes. Adult worms pairs (male and female) were incubated in a 24-well culture plate (Techno Plastic Products, TPP, St. Louis, MO, USA), containing the same medium supplemented with 10% heat-inactivated calf serum (Gibco BRL) at 37°C in a 5% CO_2_ atmosphere. For the* in vitro* test with* S. mansoni*, AA and TP were evaluated at concentrations of 12.5, 25, 50, 100, and 200 *μ*g/mL, according to procedures previously described [20, 21]. Isolated compounds from AA and TP were evaluated at 100 *μ*M. The active compound (parthenolide) was also evaluated at additional concentrations of 6.25, 12.5, 25, and 50 *μ*M. Samples were added to the culture from a 4000 *μ*g/mL stock solution in RPMI 1640 containing dimethyl sulfoxide (DMSO). The final volume in each well was 2 mL. Control worms were assayed in RPMI 1640 medium and RPMI 1640 with 0.5% DMSO as negative control groups and PZQ (5 *μ*M) as positive control group. All experiments were performed in triplicate and were repeated at least two times. Parasites were maintained for 48 h and monitored every 24 h using a light microscope in order to evaluate their general condition: motor activity, mortality rate, and tegumental alterations [3, 8].

### 2.4. Tegumental Changes

Tegumental alteration and quantification of the number of tubercles were performed for parthenolide (at concentrations of 12.5, 25, and 50 *μ*M) using a confocal laser scanning microscope. After the established times or in the occurrence of death, the parasites were fixed in formalin-acetic acid-alcohol solution (FAA) and analyzed under a confocal microscope (laser scanning microscopy, LSM 510 META, Zeiss) at 488 nm (exciting) and 505 nm (emission) [3]. A minimum of three areas of the tegument of each parasite were assessed. The numbers of tubercles was counted in 20,000 *μ*m^2^ of area calculated with the Zeiss LSM Image Browser software.

### 2.5. Viability Assay

Mammalian Vero cells (African green monkey kidney fibroblast) used in this study were obtained from the American Type Culture Collection (ATCC CCL-81; Manassas, VA) and provided by Dr. Ronaldo Z. Mendonça (Laboratório de Parasitologia, Instituto Butantan, São Paulo, Brazil). Cytotoxicity was determined as previously described [22] using different concentrations of parthenolide (25, 50, 100, and 200 *μ*M).

### 2.6. Statistical Analysis

The statistical tests were performed with the GRAPHPAD PRISM (version 6.0) software. Significant differences were determined by one-way analysis of variance (ANOVA) and applying Tukey's test for multiple comparisons with a level of significance set at *P* < 0.05. IC_50_ values were also calculated using sigmoid dose-response curves in aforementioned software [9].

## 3. Results and Discussion

Schistosomiasis is a neglected tropical disease, in which there is no vaccine and chemotherapy relies only on PZQ [5]. In this scenario, the search for bioactive plant extracts, which can be used as anthelmintic, has received considerable attention [2, 6, 8, 11]. Among medicinal plants,* A. absinthium* and* T. parthenium* have shown to be good sources for providing biologically activity compounds against parasites [13, 14, 16]. Then, as part of our search for schistosomicidal molecules from Brazilian plants [2, 3, 8, 11], we performed, for the first time, the* in vitro *schistosomicidal assay of the crude extracts of* A. absinthium* (AA) and* T. parthenium* (TP).

As shown in Table 1, AA (200 *μ*g/mL) causes 100% mortality in all adult parasites, as well as tegumental alterations and significant decrease in motor activity. In addition, after 48 h of incubation, with AA (100 and 50 *μ*g/mL), all worm pairs were dead, also showing tegumental alterations and significant decrease in motor activity (Table 1). However, at 25 and 12.5 *μ*g/mL, AA was inactive (Table 1). Dichloromethane extracts from* Artemisia* spp. have been assessed against several parasites. Mojarrab et al. [23] evaluated the dichloromethane extracts of* A. armeniaca* and* A. aucheri*, showing pronounced* in vitro* antimalarial activity for both extracts, whose effects were associated with high content of sesquiterpene lactones.

**Figure 1:**
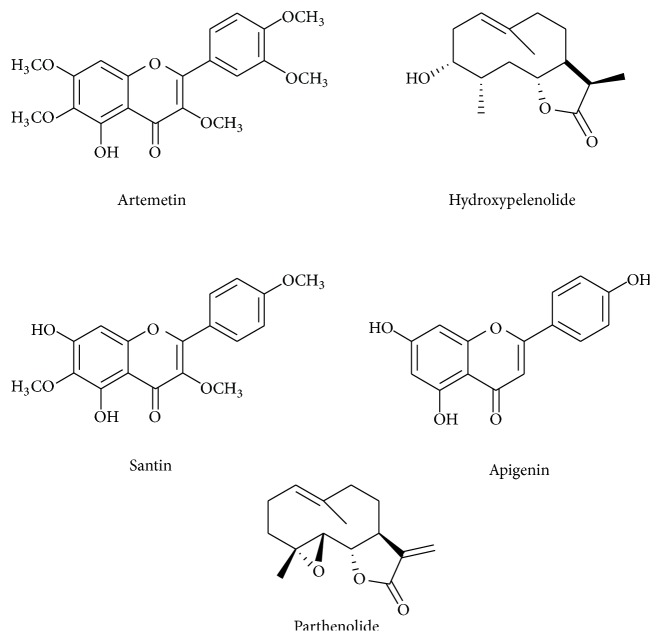
Chemical structures of compounds isolated from crude extracts of* A*.* absinthium* L. and* T. parthenium*.

Then, AA was submitted to chromatographic fractionation, yielding two pure compounds (Figure 1), which were chemically identified by ^1^H- and ^13^C-NMR data analysis in comparison to literature as artemetin [24] and hydroxypelenolide [25]. After that, the isolated compounds (artemetin and hydroxypelenolide) were also evaluated against adult worms of* S. mansoni* (Table 2). However, neither artemetin nor hydroxypelenolide was active against schistosomes when tested at 100 *μ*M.

Similarly, the* in vitro* schistosomicidal effects of TP against adult worms of* S. mansoni* were also evaluated. As shown in Table 1, TP showed schistosomicidal activities at 200, 100, and 50 *μ*g/mL, causing death of 100% of adult worms after 24 h of incubation, as well as significant tegumental alterations and decrease in motor activity. However, TP was inactive at 25 and 12.5 *μ*g/mL (Table 1). In addition, positive control (PZQ, 5 *μ*M) resulted in the death of all parasites within 24 hours, whereas no mortality was observed in the worms of the negative (RPMI medium) and solvent control (RPMI medium plus 0.5% DMSO) groups.

Chromatographic fractionation of TP led to the isolation of three compounds (Figure 1), which were chemically identified by ^1^H- and ^13^C-NMR data analysis in comparison to literature: santin [17], apigenin [17], and parthenolide [16]. Purity of all isolated compounds was estimated to be higher than 95% by HPLC-DAD analysis.

In a preliminary screening (Table 2), isolated compounds from TP were tested at 100 *μ*M against* S. mansoni* adult worms. Among isolated compounds, parthenolide (Figure 1) was active, causing 100% mortality, tegumental alterations, and reduction in motor activity of all adult worms of* S. mansoni *after 24 hours of* in vitro* drug exposure. On the other hand, santin and apigenin were inactive.

In this regard, previous studies have associated the antiparasitic activities of* T. parthenium*, such as leishmanicidal [16] and trypanocidal [15, 18] activity, with sesquiterpene lactones. When analyzed at lower concentrations, investigations revealed that all adult worms were killed by parthenolide at 50, 25, and 12.5 *μ*M, while no activity was found at concentration of 6.5 *μ*M, even after 72 h of incubation (Table 2). The concentration of parthenolide required to kill 50% (LC_50_) of adult worms* in vitro* was 9.5 *μ*M in 72 h. In addition, the incubation of schistosomes with parthenolide kept the male and female adult worms separated, which prevented the mating process and further oviposition. In contrast, all of the worms of the control group remained paired and egg production was observed.

**Figure 2:**
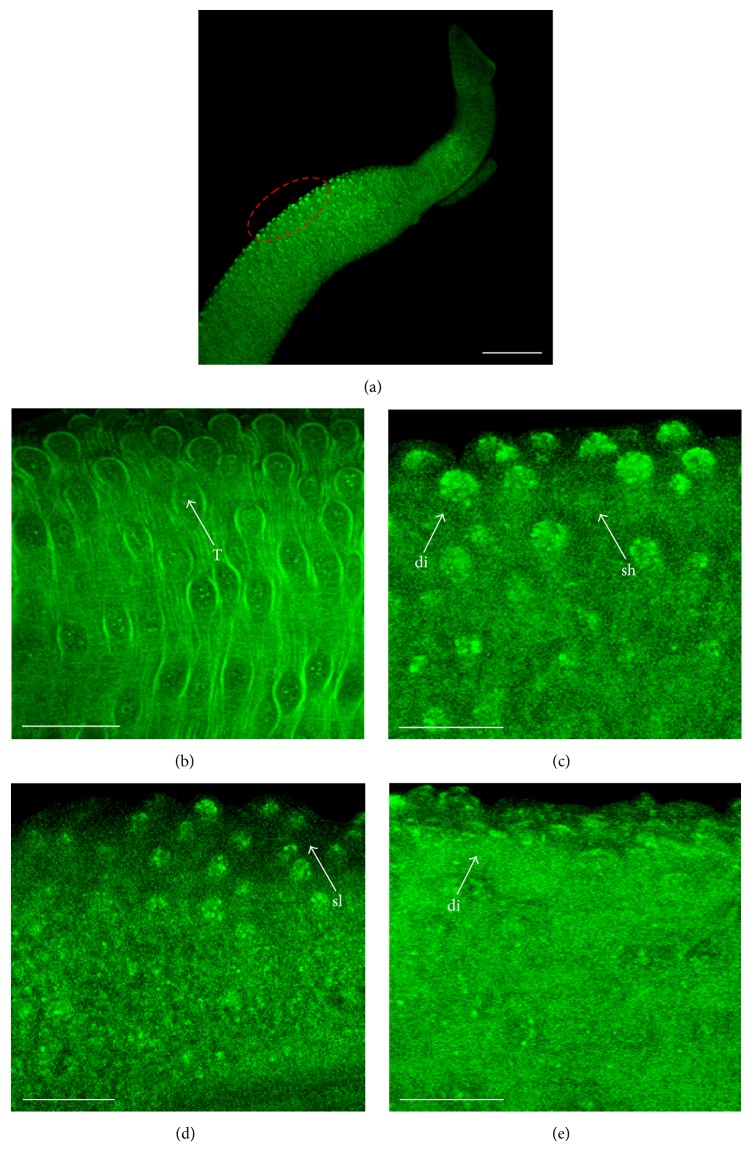
Confocal laser scanning microscopy observations of* S. mansoni* male worms after* in vitro *incubation with parthenolide. Adult worms were incubated in 24-well culture plates containing RPMI 1640 medium with 0.5% DMSO and treated with parthenolide at different concentrations. (a) General view of the anterior worm region showing, in red, the location where tegument was analyzed. (b) Control containing RPMI 1640 with 0.5% DMSO, showing tubercles (T). (c) 12.5 *μ*M parthenolide, showing tubercles shrunken (sh) and disintegrated (di). (d) 25 *μ*M parthenolide, showing surface sloughing (sl). (e) 50 *μ*M parthenolide, showing tubercles disintegrated (di). (b, d) Bars = 200 *μ*m. (c, e) Bars = 50 *μ*m.

Because parthenolide was active against adult schistosomes, we further analyzed its effects on* S. mansoni* tegument. Light microscopic investigations demonstrated that parthenolide (12.5, 25, and 50 *μ*M) caused morphological alterations in the tegument of schistosomes (Table 1). To further describe the effects of parthenolide on tegument, we performed an analysis using confocal laser scanning microscopy. As shown in Figure 2, morphological alterations of the tegument on the* S. mansoni *surface were detected with parthenolide at 12.5 *μ*M (Figure 2(c)), 25 *μ*M (Figure 2(d)), and 50 *μ*M (Figure 2(e)). Meanwhile, no abnormality was seen in the worms maintained in the negative control group. Thus, a pattern consisting of a combination of changes in the surface morphology was detected and correlated to the death of the adult worms. These pronounced changes in the aspect of tubercles, which often appeared collapsed and disrupted, were similar to those reported in studies with other isolated natural compounds, such as piplartine, (+)-limonene epoxide, cardamonin, and licoflavone B [2, 3, 8, 21, 22].

**Figure 3:**
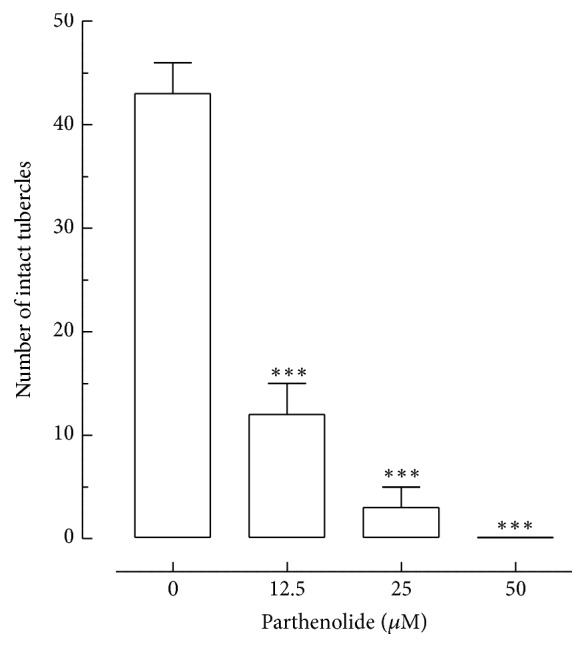
Effect of parthenolide on tubercles of* S. mansoni* male worms. The quantification of the number of tubercles was performed using confocal microscopy. Indicated are numbers of intact tubercles and these numbers were measured in a 20,000 *μ*m^2^ of area calculated with the Zeiss LSM Image Browser software. Praziquantel (PZQ, 5 *μ*M) was used as positive control. A minimum of three tegument areas of each parasite were assessed. Values are means ± SD (bars) of ten male adult worms. ^*∗∗∗*^ *P* < 0.001 compared with untreated groups.

Additionally, morphological alterations on* S. mansoni* tegument were also quantitatively analyzed by counting the tubercles on the dorsal surface of male parasites after exposure to different concentrations of parthenolide (Figure 3). As shown in Figure 3, parthenolide caused disintegration on tubercles of* S. mansoni* male worms. For example, the number of intact tubercles in an area of 20 000 *μ*m^2^ on male worms of the negative control was 43, while in the groups exposed to 12.5, 25, and 50 *μ*M of parthenolide it was, respectively, 12, 3, and 0,1. Similar results were obtained from the paired schistosomes exposed to some natural compounds, such as licoflavone B [8] and (+)-limonene epoxide [21]. The tegument of schistosomes is usually considered for its key role in nutrient uptake, secretory functions, and parasite protection [22], being a major target for the development of antischistosomal drugs [2, 3, 8]. Our results clearly show that parthenolide led to a pronounced change in the aspect of the tubercles, which often appeared shrunken and disrupted, as well as sloughing in the tegumental surface. Thus, the death of adult worms caused by parthenolide could be associated with its effects on tegumental surface of adult schistosomes.

Furthermore, our results showed that parthenolide is nontoxic to the mammalian Vero cells at concentrations that effectively kill the adult worms of* S. mansoni *(data not shown), giving support to its potential as lead compound for the development of novel therapeutic schistosomicidal drug.

Several studies have demonstrated the antiparasitic activity of sesquiterpene lactones isolated from* T. parthenium* [16, 26]. Tiuman et al. [16] found that parthenolide is the most abundant sesquiterpene lactone found in* T. parthenium*, showing significant antileishmanial activity. In addition, parthenolide also showed to be effective against forms of* Trypanosoma cruzi* [15].

The mechanism by which parthenolide exerts its* in vitro* schistosomicidal effect is unclear. However, it is reported that the antiparasitic activity of sesquiterpene lactones is mediated chemically by *α*,*β*-unsaturated carbonyl structures, such as an *α*-methylene-*γ*-lactone found in parthenolide [26, 27]. This structural group could react with nucleophiles, especially sulfhydryl groups of cysteine, by a Michael-type addition. Therefore, considering that in the tegument surface and tubercles of schistosomes there are several cysteine residues, parthenolide could react with those exposed sulfhydryl groups, causing morphological alterations and inhibiting a variety of enzymes in schistosomes tegument [28, 29]. Also, these chemical alkylating properties are not found in hydroxypelenolide (Figure 1), which could explain its inactivity* in vitro* against adult schistosomes. In this regard, Izumi et al. [15] demonstrated that parthenolide induced alterations in the body shape and loss of integrity of the plasma membrane in trypomastigote forms of* Trypanosoma cruzi*. Also, it is reported that the antileishmanial action of parthenolide is associated with a loss of membrane integrity and mitochondrial dysfunction [30]. Considering that parthenolide possesses a wide pharmacological potential and low toxicity [31, 32], our findings open the route to further schistosomicidal studies with this compound.

## 4. Conclusion

The present study has demonstrated the* in vitro* schistosomicidal activity of the crude extracts of* T. parthenium* and* A. absinthium*. In addition, we demonstrated, for the first time, that parthenolide (isolated from* T. parthenium*) is active* in vitro* against adult worms of* S. mansoni*, causing damage in the worm's tegument. Considering the obtained results, parthenolide is a promising compound that could be evaluated in additional* in vivo* schistosomicidal investigations.

## Figures and Tables

**Table 1:** *In vitro* effects of crude extracts of *A. absinthium* (AA) and *T. parthenium *(TP) against *S. mansoni *adult worms.

Groups	Incubation period (h)	Dead worms (%)^a^	Decrease of motor activity (%)^a^		Worms with tegumental alteration (%)^a^	
			Slight	Significant		Slight
Control^b^	24	0	0	0	0	0
	48	0	0	0	0	0
	72	0	0	0	0	0

0.5% DMSO	24	0	0	0	0	0
	48	0	0	0	0	0
	72	0	0	0	0	0

PZQ^c^	24	100	0	100	0	100
	48	100	0	100	0	100
	72	100	0	100	0	100

AA						
200 *µ*g/mL	24	100	0	100	0	100
	48	100	0	100	0	100
	72	100	0	100	0	100

100 *µ*g/mL	24	0	0	0	0	0
	48	100	0	100	0	100
	72	100	0	100	0	100

50 *µ*g/mL	24	0	0	0	0	0
	48	100	0	100	0	100
	72	100	0	100	0	100

25 *µ*g/mL	24	0	0	0	0	0
	48	0	0	0	0	0
	72	20	0	20	0	20

12.5 *µ*g/mL	24	0	0	0	0	0
	48	0	0	0	0	0
	72	0	0	0	0	0

TP						
200 *µ*g/mL	24	100	0	100	0	100
	48	100	0	100	0	100
	72	100	0	100	0	100

100 *µ*g/mL	24	100	0	100	0	100
	48	100	0	100	0	100
	72	100	0	100	0	100

50 *µ*g/mL	24	100	0	100	0	100
	48	100	0	100	0	100
	72	100	0	100	0	100

25 *µ*g/mL	24	0	0	0	0	0
	48	0	0	0	0	0
	72	50	0	50	0	50

12.5 *µ*g/mL	24	0	0	0	0	0
	48	0	0	0	0	0
	72	0	0	0	0	0

^a^Percentages relative to 20 worms investigated.

^b^RPMI 1640.

^c^Tested at concentration of 5 *µ*M.

**Table 2:** *In vitro* effects of isolated compounds against *S. mansoni *adult worms.

Groups	Incubation period (h)	Dead worms (%)^a^	Decrease of motor activity (%)^a^		Worms with tegumental alteration (%)^a^	
			Slight	Significant		Slight
Control^b^	24	0	0	0	0	0
	48	0	0	0	0	0
	72	0	0	0	0	0

0.5% DMSO	24	0	0	0	0	0
	48	0	0	0	0	0
	72	0	0	0	0	0

PZQ^c^	24	100	0	100	0	100
	48	100	0	100	0	100
	72	100	0	100	0	100

Artemetin						
100 *µ*M	24	0	0	0	0	0
	48	0	0	0	0	0
	72	0	0	0	0	0

Hydroxypelenolide						
100 *µ*M	24	0	0	0	0	0
	48	0	0	0	0	0
	72	0	0	0	0	0

Santin						
100 *µ*M	24	0	0	0	0	0
	48	0	0	0	0	0
	72	0	0	0	0	0

Apigenin						
100 *µ*M	24	0	0	0	0	0
	48	0	0	0	0	0
	72	0	0	0	0	0

Parthenolide						
100 *µ*M	24	100	0	100	0	100
	48	100	0	100	0	100
	72	100	0	100	0	100

50 *µ*M	24	100	0	100	0	100
	48	100	0	100	0	100
	72	100	0	100	0	100

25 *µ*M	24	100	0	100	0	100
	48	100	0	100	0	100
	72	100	0	100	0	100

12.5 *µ*M	24	0	0	0	0	0
	48	100	0	100	0	100
	72	100	0	100	0	100

6.25 *µ*M	24	0	0	0	0	0
	48	0	0	0	0	0
	72	0	0	0	0	0

^a^Percentages relative to 20 worms investigated.

^b^RPMI 1640.

^c^Tested at concentration of 5 *µ*M.
